# Characterization of the complete plastome of *Atropanthe sinensis* (Solanaceae)

**DOI:** 10.1080/23802359.2019.1662742

**Published:** 2019-09-06

**Authors:** Zhilin Jiang, Ziyoviddin Yusupov, Xianhan Huang, Dilmurod Makhmudjanov, Komiljon Tojibaev, Tao Deng

**Affiliations:** a Puer University, Institute of Agricultural and Garden Technology, Puer, China;; b Key Laboratory for Plant Diversity and Biogeography of East Asia, Kunming Institute of Botany, Chinese Academy of Sciences, Kunming, China;; c University of Chinese Academy of Sciences, Beijing, China;; d National Herbarium of Uzbekistan, Institute of Botany, Academy Sciences of Uzbekistan, Tashkent, Uzbekistan

**Keywords:** Chloroplast genome, phylogenetic analysis, *Atropanthe sinensis*

## Abstract

*Atropanthe sinensis* Pascher (genus *Atropanthe,* family Solanaceae) is an endangered and medical important plant of China. Here, we report the complete plastome of *A. sinensis*, which was found to be 156,565 bp in length and encodes 114 annotated known unique genes, including 80 protein-coding genes, 4 ribosomal RNAs, and 30 transfer RNAs. Among them, **10** coding genes contained one intron each and two genes (*clpP* and *ycf3*) contained two introns each. The result of the phylogenetic analysis showed that genera *Scopolia* and *Przewalskia* were placed as sister groups to the *Atropanthe.*

The genus *Atropanthe* Pascher is monotypic and naturally distributed in China (Zhang et al. 1994). *Atropanthe sinensis* is an important medicinal plant as its root is used as a medicine for relieving pain. *Atropanthe sinensis* can be easily distinguished from other species of Solanaceae by corolla zygomorphic, funnel-shaped, and calyx lobes alike (Zhang et al. 1994). Here, we assembled and characterized the first complete plastome of genus *Atropanthe*.

Fresh leaves of *A. sinensis* were collected from Wufeng, Hubei province, China (E110°33′41″, N30°15′18″). A voucher specimen of *A. sinensis* was deposited in the Herbarium of Kunming Institute of Botany, Chinese Academy of Sciences (KUN, Deng4216). Total DNA was extracted according to the modified CTAB method (Doyle and Doyle, 1987), then fragmented into150 bp for library construction and sequenced using an Illumina HiSeq 2500 system at BGI (Shenzhen, Guangdong, China). We assembled the cp genome based on the methods of Jin et al. (2018), and the plastome of *Scopolia parviflora* was used as a reference genome (Genbank accession: NC_030282). We annotated *A. sinensis* used Geneious v10.2 (Kearse et al. 2012), then start and stop codons and intron/exon boundaries were manual edited. The complete chloroplast genome of *A. sinensis* has been deposited into the GenBank (Genbank accession: MK411818). Phylogenetic analysis of *A. sinensis* with 20 Solanaceae species was performed using RAxML-HPC BlackBox v8.1.24 software (Stamatakis 2006) with the GTRGAMMAI model. The result of the phylogenetic analysis showed that genera *Scopolia* and *Przewalskia* were placed as sister groups to the genus *Atropanthe* (Figure 1).

**Figure 1.**
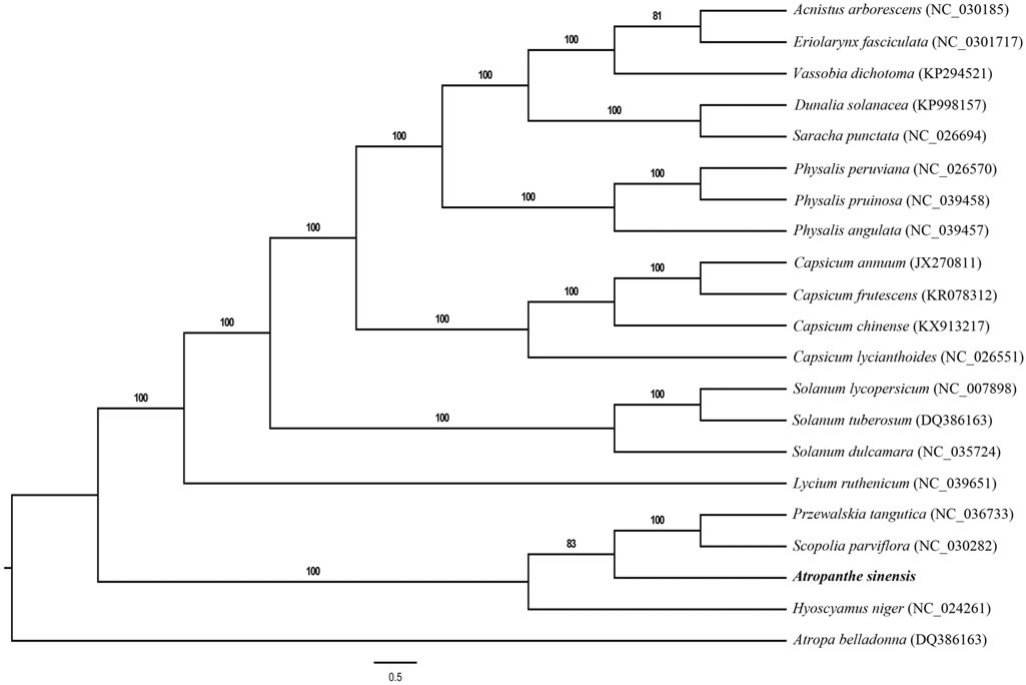
The maximum likelihood (ML) tree of sampled species of Solanaceae. Numbers associated with branches are ML bootstrap support values.

The complete cp genome sequence of *A. sinensis* is 156,565 bp and presents a typical circular structure, including a pair of IRs (25,939 bp, GC: 42.8% for each) that the genome divided into two single-copy regions (LSC 86,600 bp, GC: 35.6%; SSC: 18,087 bp, GC: 31.9%). A total of 114 genes were identified in the *A. sinensis* genome, including 80 protein-coding genes, 34 tRNA genes, 4 ribosomal RNA genes. Among these, 10 coding genes contained one intron each and two genes (clpP and ycf3) contained two introns. Our reported cp genome of *A. sinensis* will be useful for further population genomic studies, phylogenetic analyses, and genetic engineering studies of family Solanaceae.
